# Roles of planar cell polarity pathways in the development of neutral tube defects

**DOI:** 10.1186/1423-0127-18-66

**Published:** 2011-08-24

**Authors:** Gang Wu, Xupei Huang, Yimin Hua, Dezhi Mu

**Affiliations:** 1 Department of Pediatrics, West China Second University Hospital, Sichuan University, Chengdu, Sichuan 610041, China; 2 Department of Biomedical Science, College of Medicine, Florida Atlantic University, Boca Raton, FL 33431, USA; 3 Department of Neurology, Newborn Brain Research Institute, University of California, San Francisco, CA94143, USA

**Keywords:** Neural tube defects, planar cell polarity, organ morphogenesis, signaling pathway

## Abstract

Neural tube defects (NTDs) are the second most common birth defect in humans. Despite many advances in the understanding of NTDs and the identification of many genes related to NTDs, the fundamental etiology for the majority of cases of NTDs remains unclear. Planar cell polarity (PCP) signaling pathway, which is important for polarized cell movement (such as cell migration) and organ morphogenesis through the activation of cytoskeletal pathways, has been shown to play multiple roles during neural tube closure. The disrupted function of PCP pathway is connected with some NTDs. Here, we summarize our current understanding of how PCP factors affect the pathogenesis of NTDs.

## Background

Neural tube defects (NTDs), arise when the neural tube, the embryonic precursor of the brain and spinal cord, fails to close during neurulation. Defects in neural tube closure are the second most common human birth defects, after congenital heart defects [1]. Recent birth prevalence estimates show that NTDs account for 0.5 per 1000 in the United States during 2001-2004, 1 to 1.5 per 1000 in Western Australia during 2001-2006, and 2.8 per 1000 in Iran during 1998-2005, while prevalence in Shanxi, a province in North China, reach to 19.9 per 1000 during 2002-2004 [2].

The cranial region (anencephaly) or the low spine (open spina bifida and myelomeningocele) are most commonly affected [3]. NTDs affecting the brain are invariably lethal perinatally, whereas open spina bifida is compatible with postnatal survival but frequently results in serious handicap, because neurological impairment below the lesion leads to lack of sensation, inability to walk and incontinence [4].

### Neural tube formation and NTDs classification

Neural tube closure is the result of neurulation, a process in which the neural plate bends upwards and eventually fuses to form the hollow tube that will become the brain and the spinal cord. The driving force of neural tube closure is provided and maintained by cells undergoing convergence and extension (CE) [5].

Both fish (such as zebrafish) and amphibian (such as Xenopus) embryos require this process [6,7]. Neurulation is conserved between mammalian species [8] and can be conventionally divided into primary and secondary phases [9].

In primary neurulation, the fusion occurs along the spine and culminates in final closure at the posterior neuropore. Closure is initiated at the hindbrain/cervical boundary (Closure 1) and then spreads bi-directionally into the hindbrain and along the spinal region. Separate closure initiation sites occur at the midbrain-forebrain boundary (Closure 2) and at the rostral extremity of the forebrain (Closure 3). However, Closure 2 found in mice may be absent from human neurulation [10].

The secondary phase occurs at lower sacral and caudal levels, where the neural tube is formed in the tail bud without neural folding [4,11].

Failure of Closure 1 leads to the most severe NTD, craniorachischisis, which combines an open neural tube encompassing the midbrain, hindbrain and entire spinal region. If Closure 1 is completed but closure of the cranial neural tube is incomplete, anencephaly develops, with cases exhibiting either defects confining in the midbrain (meroanencephaly) or lesions extending into the hindbrain (holoanencephaly) [12]. Failure of Closure 3 is uncommon but, when present, yields split face with anencephaly. In the spinal region, failure of final closure at the posterior neuropore yields open spina bifida (also called myelocele or myelomeningocele), in which the upper limit can be of varying axial level [9]. By contrast, defective secondary neurulation leads to 'closed' forms of spina bifida [9].

### Human NTDs and possible causes

Epidemiological studies provide an opportunity to identify risk factors for NTDs, such as dietary or teratogenic agents, to which susceptibility may be modified by genetic predisposition [3,13,14]. Identification of causative factors is confounded by the fact that the majority of these malformations appears to result from a combination of genetic and non-genetic factors (environmental contributions) [3].

Many non-genetic factors may be associated with NTDs formation. They include: parental socioeconomic status [15,16], parental age [17], parental race [18], hyperthermia during early pregnancy [19], maternal health (such as diabetes [20], obesity [21]), dietary agents or maternal nutrition (such as the uptake of folate [22-24], inositol [25,26]), chemical teratogenic agents (such as valproic acid [27], retinoic acid [28], trichostatin A [29], exposure to pesticides [30] and selective serotonin-reuptake inhibitors [31] and so on).

As for genetic factors, the cumulative number of reported mouse genetic mutants with NTDs continues to rise steadily, from approximately 200 in 2007 [32] to approximately 245 in 2010 [33]. The different mouse gene mutations, naturally occurring or targeted mutations, are associated with various NTD phenotypes [3,9,32]. Many of the NTD-causing mouse mutations implicate specific signaling pathways such as PCP signaling, Sonic hedgehog (Shh) signaling, BMP signaling, Notch signaling, retinoid signaling and inositol metabolism [4]. Those signaling pathways are involved in the maintenance of the cell cycle, the regulation of the actin cytoskeleton, chromatin organization and epigenetic modifications including methylation and acetylation [3].

However, although there is evidence for a strong genetic component in the individual liability to NTDs in humans, little is known about the nature of these risk genes about their interactions with each other. In general, the risk genes are present in the affected individuals. However, it is unknown whether the same risk genes are shared by all population [33].

Meanwhile, gene-dosage can also affect neural tube closure. Chromosomal abnormalities, especially trisomy 13 and 18, are strongly associated with central nervous system malformations [34,35], and a gene dosage imbalance of 16q12.1-q22.1 is also associated with spina bifida in the patient [36].

Recently, a major advance in understanding of the genetic basis of neurulation is the finding that the initiation of Closure 1 requires noncanonical Wnt signaling, the so-called planar cell polarity (PCP) signaling pathway [3].

### PCP signaling pathway

PCP, which is within the plane of an epithelium, is not restricted to epithelial tissues, but is also found in mesenchymal cells during animal development [5].

There are two evolutionarily conserved sets of PCP factors that act together to coordinate PCP establishment: the Frizzled (Fz)/Flamingo (Fmi) core genes and the Fat/Dachsous (Ds) PCP system [5].

In Fat/Ds system, Dachsous (Ds) and Fat (Ft), together with a transmembrane Golgi complex protein, Four-jointed (Fj), set up a global polarity signal, which is then sensed and propagated by the asymmetric assembly of cell-surface complexes, transmitting signal between cells [37-40]. Members of the Fat/Ds group are expressed in gradients and their graded expression is under the control of canonical Wg-signaling [41,42]. It has been suggested that Fat/Ds acts upstream of Fz/PCP signaling, largely based on data on the fly eye [39,41]. However, recent genetic mosaic experiments in the *Drosophila *abdomen argue that these two systems may function in parallel rather than in series [43]. As there is no report about the relationship between Fat/Ds system and NTDs, in this review, we will not discuss the system in detail.

The Fz/Fmi system is the principal PCP signaling pathway and appears to be the "noncanonical" Wnt signaling pathway [44]. The components of the Fz/Fmi system include transmembrane proteins, such as Frizzled (Fz), Flamingo (Fmi, Celsr1 in in human and rodents), Strabismus/Van Gogh (Stbm/Vang) and intracellular proteins, such as Dishevelled (Dsh in *Drosophila*; Dvl in vertebrates), Prickle (Pk), and Diego (Dgo; Diversin in vertebrate and inversin in mouse). Scribble (Scrib) [45,46] and Ptk [47,48] are sometimes regarded as PCP proteins. All the components work together, through either coordination or antagonism. For example, Vang/Pk is thought to antagonize the Fz/Dvl signaling [49,50].

The PCP system, to which Wnt5a and Wnt11 have been clearly linked in vertebrates, is related to the canonical Wnt signaling pathway, which interprets the directional signal to produce subcellular asymmetries [37,44,51-54] Downstream of the PCP system are so-called 'PCP effector', which are the novel proteins, Inturned, Fuzzy and Fritz [55,56]. They mediate the PCP signaling in different tissues. This system can play an important role in polarized cell movement (cell migration) and organ morphogenesis through the activation of cytoskeletal pathways, such as the small GTPases RhoA and cdc42, Rho kinase, protein kinase C (PKC) and Jun N-terminal kinase (JNK) 1 [51,57]. Activation of the PCP signaling in a given cell population is able to exert changes in neighboring cells that do not express PCP elements [58].

### The role of PCP signaling pathway in NTDs

The genetic and molecular dissection of PCP began 29 years ago with the realization by Gubb and Garcia-Bellido that a small set of genes controls the polarity of cuticular hairs and bristles in *Drosophila *[44,59].

At that time many vertebrate tissues and developmental processes have been shown to display typical PCP features [51,60,61]. Time-lapse studies in Xenopus revealed that PCP-dependent CE was required to narrow the distance between the elevating neural folds, allowing their apposition and fusion [62]. Other analyses in Xenopus [63,64], zebrafish [65,66] and mouse [67] also show that the PCP factors are key players in the process of CE movement during gastrulation and neurulation.

For a more detailed understanding of the PCP pathway in zebrafish gastrulation, Gong observed that PCP pathway plays a conserved role in vertebrate axis elongation, orienting both cell intercalation and mitotic division [68]. However, Ciruna *et al *have shown that PCP pathway is required for the reintegration of newly postmitotic cells into the neuroepithelium [69]. They also observed that loss of Vangl2 (*trilobite*) leads to an accumulation of apical daughter cells from recent mitoses in the center of the U-shaped, and incompletely closed, neural fold [69]. A striking demonstration that the failure to reintegrate these cells underlies the neural tube closure defect came from the observation that pharmacologically blocking cell division in the *trilobite *mutant late in gastrulation restores neural tube closure, presumably because without cell division there are no extruded cells [69]. By contrast, mitotic inhibitors did not rescue the CE phenotype caused by the *trilobite *mutation [44].

For the spatio-temporal expression, PCP is believed to initiate Closure 1 in mice [3]. In another perspective, the PCP pathway is believed to be responsible for caudal NTDs, though *Dvl2^-/- ^*mice also display some rostral defects [5,70], while the Shh pathway accounts for most of the rostral defects [5,55]. However, in Patched1 null mice, both rostral and caudal defects are seen [71], suggesting that both pathways act at different stages during neurulation. When Shh pathway regulates neural plate bending and specification of the ventral neural cell fates, the PCP pathway drives neural tube closure [72].

### PCP protein mutations and NTDs

When the correct expressivity of proteins in PCP signaling is disturbed, caused either by environmental factors or by genetic factors, some NTDs can occur.

#### Frizzled (Fz)

*Fz*, the first PCP gene to be defined molecularly, and also a member of the Wnt receptor family, codes seven transmembrane helices [73] and an amino-terminal cysteine-rich domain (CRD) that is sufficient and necessary for binding with the ligands of the Wnts [74-76]. It can also bind Dsh and recruit Dsh and Dgo to the membrane. In mammals, Fz genes have been implicated in a variety of developmental processes, including the nervous system formation. Fz3 is required for axonal outgrowth and guidance in the CNS [77,78]. Fz3 can also play a role during sympathetic neuron development via the activation of β-catenin [79].

During the gastrulation in Xenopus, overexpression of *Fz7 (Xfz7) *in the dorsal equatorial region affects the CE movement and causes a delay of the mesodermal development [80]. In the mouse, Fz3 and Fz6 play a role in neural tube closure. *Fz3^-/-^; Fz6^-/- ^*embryos exhibit craniorachischisis with nearly 100% penetrance, and these mice die within minutes after birth [81]. *Fz1 *and/or *Fz2 *mutations can cause defects in neural tube closure [82].

#### Flamingo (Fmi)/Starry night(Stan)/Celsr1

Three Fmi gene orthologs in human and rodents are named *celser1 - celser3 *respectively. Fmi genes encode proteins of the cadherin superfamily which are seven transmembrane proteins with nine cadherin repeats in the extracellular domain, and an uncharacterized intracellular C terminus. The *Drosophila *Fmi gene regulates epithelial planar cell polarity and dendritic field deployment [83,84]. Recent studies show that the primary function of Fmi is to participate in the asymmetry of PCP [85,86]. In mouse, the homozygous *Celsr1 *mutants (*Crsh *and *Scy*) exhibit severe neural tube defects, such as craniorachischisis, as a result of failure to initiate neural tube closure, providing evidence for the function of the Celsr family that are involved in a planar cell polarity pathway in vertebrate neurulation [87].

#### Strabismus (Stbm)/Van Gogh (Vang)/vangl

*Vangl1 *and *Vangl2 *are mammalian homologs of *Drosophila *gene *Van Gogh (Vang)*, also known as *Strabismus *in which mutations disrupt the organization of various epithelial structures, causing characteristic swirled patterns of hairs on wing cells and misorientation of eye ommatidia [88]. Exon-intron structure of mammalian *Vangl1 *and *Vangl2 *orthologs was well conserved [89]. *Vangl2 *encodes a membrane protein comprising four transmembrane domains and a large intracellular domain with a PDZ-domain-binding motif at its carboxy terminus [90].

Vangl2 can modulate actin cytoskeleton through the small GTPases RhoA and Rac and the downstream Rho kinase. Thus it is partially responsible for a variety of changes in cell adhesion, polarity, and short-range tissue movements [91].

Studies of Stbm genes and the proteins that they encode in mice, flies, frogs and fish have shown that they have a crucial role in regulating planar cell polarity and convergent extension movements [88]. In fly mutated embryos, the polarity of the ommatidia of the compound eye and the hairs of the wing and thorax are disrupted, such that rather than pointing in the same direction, they point in multiple directions [92]. In zebrafish, *trilobite *mutant embryos (loss of *Stbm*) have defects in gastrulation movements and posterior migration of hindbrain neurons [65], resulting in ectopic neural progenitor accumulations and NTDs [69]. In Xenopus, the homolog of *Stbm *is called *xstbm*. The *xstbm *can regulate convergent extension in both dorsal mesoderm and neural tissue by either increasing or decreasing the *Vangl2 *function due to its optimal retard of convergent extension movements [93]. Reduction of *xstbm *function using a morpholino antisense oligo also causes the trunk shortening [94].

Loop-tail *(LtapLp*, also called as *Lp, Ltap, Lpp1*) gene is a semidominant mutation that affects neurulation in mice, which are characterized by a looped-tail appearance (pig tail) and wobbly head movements while homozygous embryos exhibit a neural tube closure defect that extends from the caudal midbrain to the tip of the tail [95]. A potential role of PCP in NTDs came to light following positional cloning of *Vangl2 *in the loop-tail mouse mutants that exhibit a severe NTD, craniorachischisis [90,96]. Subsequently, several studies have shown that *Vangl2 *can also interact with different genes and cause several forms of NTDs. For example, *Dvl3^+/-^; LtapLp^/+ ^*can cause craniorachischisis or exencephaly. *Dvl3*^-/-^; *Ltap*^*Lp*/+ ^mutants cause craniorachischisis [97]. Genetic interaction between *Wnt5a *and *Ltap/Vangl2 *could enhance the penetrance of neural tube closure and all *Wnt5a^-/-^; LtapLp^/+ ^*mice exhibited craniorachischisis [98]. Sequence analysis has not been success thus far in identifying the mutations in human Vangl2 gene in patients with craniorachischisis [99], although the *Vangl2 *mutation was identified in stillborn or miscarried fetuses with neural-tube defects [100].

However, the mutation in *Vangl1 *was found in patients with familial and sporadic NTDs, who exhibited a caudal neural tube, including craniorachischisis. Furthermore, the result showed that the *Vangl1 *mutations disrupted the physical interaction with Dvl [101]. These data indicate that *Vangl1 *is a risk factor in human neural-tube defects. Later, mutations in *Vangl1 *were detected in spinal dysraphisms, providing further evidences to support the role of *Vangl1 *as a risk factor in the development of spinal NTDs [102].

#### Disheveled (Dsh/Dvl)

Disheveled proteins are important signaling components in both the canonical β-catenin/Wnt pathway [103], and the PCP pathway [97]. It is a cytoplasmic protein containing DIX, PDZ, DEP domains and is recruited to membrane by Fz, undergoing extensive phosphorylation. Homologues of Disheveled are Xdsh in Xenopus, and Dvl1, Dvl2 and Dvl3 in vertebrate. Disheveled is highly conserved and play an important role in CE movement. In PCP pathway, Disheveled acts in the downstream of Wnt11 and Wnt5a and the upstream of Ca^2+^/CamKII, JNK, and the Rho GTPase family members RhoA, Rac1, and Cdc42 [104].

In vertebrate, Dvl1, Dvl2 and Dvl3 participate in the CE movement. *Dvl*1^*-/- *^[105], *Dvl3*^*-/- *^and *Dvl1^-/-^; Dvl3^-/- ^*double mutants [7] do not display neural tube defects. Mice with targeted inactivation of the Dvl1 gene were found to exhibit alterations in sensorimotor gating and social interaction [105] and *Dvl2 *does not seem to play a similar role in the same way [70]. *Dvl2^-/- ^*embryos displayed thoracic spina bifida, while virtually all *Dvl1/2 *double mutant embryos displayed a craniorachishisis, a completely open neural tube from the midbrain to the tail [7,70]. For Dvl3, which is also required for signals in the PCP pathway to regulate the CE movement during the development of the neural tube, neurulation appeared normal both *Dvl3^-/- ^*and *Ltap^Lp/+^(Vangl2/Ltap) *mutants, while defects were seen in both *Dvl3*^+/-^;*Ltap*^*Lp*/+ ^(7/22, 32%, 5 with craniorachischisis and 2 with exencephaly) and *Dvl3*^*-/-*^;*Ltap*^*Lp*/+ ^mutants (in a total of 16 mutants, 6 with craniorachischisis) [97]. These findings indicate that *Dvl2 *is the most important mammalian Dvl gene for neural tube closure and is sufficient by itself for normal neural tube closure. By contrast, *Dvl1 *and *Dvl3 *are not sufficient by themselves for a normal neural tube closure, but contribute significantly when *Dvl2 *is completely missing [7].

#### Diego (Dgo)/Diversin

Diego, comprises six ankyrin repeats and is co-localized with Flamingo at proximal/distal boundaries [106]. The homologue of Diego is Diversin in vertebrate and Inversin in mouse [44]. Diversin is also an essential component of the Wnt signaling pathway [107] and its centrosomal localization is crucial for its function in the Wnt signaling [108]. Diversin controls the balance between canonical and noncanonical Wnt signaling, with a higher diversin activity favoring PCP signaling and a lower diversin activity favoring canonical signaling [44].

In PCP pathway, Diversin act downstream of Wnt11 and Wnt5a and upstream of the small GTPases Rac and Rho [109]. In zebrafish [104] and Xenopus [62], knockdown of Diversin disrupts convegent extension. Div-ANK mRNA injection also disturbed CE in zebrafish embryos, which can be rescued by co-injection of mouse Inversin mRNA [104]. Moreover, combinations of low concentrations of *Wnt11/5a *Morpholino oligonucleotide (MO) and Div-ΔANK, which alone were virtually ineffective, acted synergistically in inducing strong CE phenotypes [104]. However, Diversin mRNA was unable to rescue the defects caused by Dishevelled lacking the DEP domain. and it's the same in reverse, although the two protein can interact [104].

#### Prickle (Pk)

Pk gene encodes a protein with a triple LIM domain and a novel domain that is present in human and murine. Caenorhabditis elegans has a homolog that is designated as PET. Three transcripts have been identified, Pk, PkM, and sple. In PCP signal pathway, Stbm/Vang and Pk antagonize Fz-Dsh activity [49,50,85]. Lack of both Pk and sple transcripts gives a phenotype that affects the whole body surface that is similar to those caused by deficiency of disheveled and Fz [110].

In zebrafish, both of homologs of Pk show a discrete and dynamic expression pattern during gastrulation. Both gain and loss of Pk1 function cause defects in convergent extension movement. In overexpression assays, Pk1 can inhibit the activation of Wnt/β-catenin signaling [111].

In Xenopus, orthologues of Pk is XPk, which expressed in tissues at the dorsal midline during gastrulation and early neurulation [112]. Both gain-of-function and loss-of-function of XPk severely perturbed gastrulation and caused a spina bifida in embryos, but no influence in mesodermal differentiation [113].

### Global polarization

The appropriate function of the PCP pathway in neurulation can ensure a normal global polarization, which not only means that the cells in the plane coordinate with each other, but also demands that the tissues develop harmoniously within the whole body. One attractive model in PCP pathway is Fat/Ds system. However this system is not involved in the development of NTDs. Recent studies show that this system, the Fz/Fmi system, and also the principal PCP signaling pathway, function in parallel [43].

#### Asymmetric arrangement

The specific, highly controlled, asymmetric arrangement of these PCP core components, appearing to be highly sensitive to the orientation of the cell's sides with respect to the global axis of the epithelium, allows the polarity of the cell to be established within the plane of the epithelium and promotes the rearrangement of the cytoskeletal components of the cell [97]. Although the asymmetric localization of some of the PCP factors has been documented in some vertebrate tissues, for example, during zebrafish gastrulation and neurulation, a complete data set and thus an equivalent model to *Drosophila *do not yet exist [5]. The asymmetric distribution of core PCP components such as Pk1 in the neural plate has recently been shown to be essential for neural tube closure [114]. Another example is that the asymmetric localization of Pk and Dsh during zebrafish convergent extension processes [115]. The fluorescent fusion proteins during dorsal mesoderm CE movement have shown that Pk localizes at the anterior cell edge, whereas Dsh is enriched posteriorly. The asymmetrical localization of Pk and Dsh observed in zebrafish gastrula is similar to their localization in fly, suggesting that noncanonical Wnt signaling defines distinct anterior and posterior cell properties to bias cell intercalations [115].

#### Wnt signaling pathway

Wnt signaling plays a critical role in a vast array of biological process, including cell proliferation, migration, polarity establishment and stem cell self-renewal [103]. Wnt5a and Wnt11 are the core members in Wnt pathway and also are clearly linked to the PCP signaling pathway. It has been reported that Wnt5a/pipetail and Wnt11/silberblick control CE movement in zebrafish embryogenesis via the PCP pathway [116-119].

Wnt11 is thought to be involved in the CE movement taking place during gastrulation and perhaps more broadly during organogenesis [120]. Zebrafish Wnt11 mutants *silberblick (Slb) *have typical convergent extension phenotypes [117]. Wnt5a can genetically interact with *Ltap/Vangl2 *to regulate neural tube closure. All *Wnt5a^-/-^;Ltap^Lp/+ ^*mutants exhibited craniorachischisis, indicating a drastic increase in penetrance as compared to the craniorachischisis phenotype displayed by *Wnt5a^-/- ^*(1 in 34) or *Ltap^Lp/+ ^*animals (0 in more than 100) [98].

Disheveled is a core component in both the PCP pathway and the Wnt pathway [103]. In zebrafish, *slb *phenotype, abnormal CE movement during gastrulation can be rescued by a truncated form of Disheveled [117]. In overexpression assays, Pk1 can inhibit activation of Wnt signaling during zebrafish CE movements of gastrulation [111].

Diversin, a homologue of Diego in vertebrate, is an essential component of the Wnt signaling pathway [107] and its centrosomal localization is crucial for its function in the Wnt signaling pathway [108]. Inversin, a homologue of Diego in mouse, can control the balance between canonical and noncanonical Wnt signaling [121]. A higher Inversin activity favors the noncanonical signaling (i.e. the PCP pathway) and a lower Inversin activity favors the canonical signaling [44].

Diversin, comprised six ankyrin repeats, can rescue CE phenotypes induced by *Wnt11/5a *MO. Also combinations of low concentrations of *Wnt11/5a *MO and Div-ΔANK, which alone were virtually ineffective, acted synergistically in inducing strong CE phenotypes, suggesting that Wnt5a and Wnt11 can control CE movement in zebrafish embryogenesis through Diversin [122].

#### Cilia

In vertebrates, many, if not all, epithelial cells have a single nonmotile cilium (the primary cilium), which is typically located in the center of the apical face of the cell [44]. Cilia are microtubule-based protrusions and are an important nexus for cellular signaling. They are apparently a critical junction between the signals that influence cell fate and the signals that influence cell movement [55].

Connections has recently been found between PCP and non-motile cilia based on the observation that several genes that affect vertebrate PCP also affect ciliary structure and/or function [123,124].

Bardet-Biedl syndrome (BBS) is a pleiotropic disorder characterized by age-related retinal dystrophy, obesity, polydactyly, renal dysplasia, reproductive tract abnormalities and cognitive impairment. It is genetically heterogeneous, with mutations identified in several BBS genes. A connection has been found between BBS genes and PCP [38,125]. 14% of *Bbs4^-/-^*mice display an open cephalic neural tube (exencephaly) [125]. MO knockdown of *BBS4 *in zebrafish leads to PCP phenotypes, including a failure of embryonic CE movement [44]. Other evidence suggesting a molecular connection between PCP and cilia comes from studies on the ciliary protein Inversin. This protein has been studied for some time in the context of cilia function, and it is also the core protein in PCP [55].

This connection between PCP signaling and a known ciliary protein became even more evident with the finding that the PCP proteins Vangl2 and DVL are localized at or near the base of cilia in vertebrate cells [125,126].

The most recent link between PCP and cilia comes from experiments with Xenopus embryos in which disruption of Inturned or Fuzzy elicited prominent rostral neural tube closure defects in addition to more caudal neural tube defects. These defects were shown to arise from failure of both PCP and Shh signaling [126]. It is clear is that several signal transduction proteins must localize to cilia for Shh signal transduction to proceed normally [127]. This suggests that Inturned and Fuzzy play a role in ciliary structure or function.

### The differences among species

Studies in *Drosophila*, zebrafish, Xenopus, mice, and human beings have revealed that similarities, as well as differences, exist in the PCP pathway and in the development of NTDs. The most important is that the principal PCP signaling pathway is highly conserved across species and tissues [5].

The numerous differences among species in anatomy, tissue types and morphogenetic processes, together with the existence of a number of distinct PCP components make it interesting to think about the difference in the development of TNDs among different species.

For example, Scrib and Ptk7, for which there is no evidence in *Drosophila *regarding a role in PCP, were associated with the PCP phenotypes in vertebrates when they were mutated, either alone and or in combination with other PCP gene mutations [128,129]. Other examples are genes such as Inturned and Fuzzy. They are considered to be the PCP effector genes in *Drosophila *and have been found to be associated with a convergent extension phenotype in frog or fish embryos [126]. The full length transcript of mouse Scrib is about 5,547 bp and encodes a putative protein containing 1,665 amino acids, which exhibits 88% homologue with human SCRB1, 44% homologue with *Drosophila *Scribble and 36% homologue with *C. elegan*s protein LET-413 [129].

Most PCP genes have only one isoform in zebrafish, Xenopus, whereas in other species such as rodents, there are often numerous isoforms (for example, 3 Dvls, 2 Vangls, 2 Prickles, 3 Celsrs, etc). Furthermore, the expression of some isoforms is not overlapped. As such, the studies on PCP generation in mice have been hampered because of the redundancy of the PCP genes. These studies require a more detailed analysis using as many tissues as possible. Double and triple knockout mouse lines are often required and the necessary involvement of these models makes investigations lengthy and tedious [44].

#### From mouse to man

At the embryonic level, the events of neurulation appear extremely similar between mice and humans. As a result, mouse models are commonly used in the research of NTDs. There are over 200 different mouse genes that result in NTD phenotypes either through naturally occurring mutations or through the targeted mutations [9,32]. Several mouse mutants involved in PCP signaling pathway for NTDs research, such as *looptail *[81,130-132], *circletai*l [129,133,134], *crash *[87,134], *dishevelled *knockout mouse [7,70,97], *BBS-*null mouse [125], *frizzled 3 *and *frizzled 6 *double mutants [81], *Sfrp1, Sfrp2*, and *Sfrp5 *compound mutant mice [135] and so on.

The human homologues of some of these mouse NTD genes have been examined in case-control association studies or directly sequenced in mutation screens, although with very few significant findings to date [3,99-102].

So we have the reason to ask whether it is appropriate to use mouse models for the studies of human NTDs [3].

First, in the process of neural tube closure, Closure 2 in mice is thought to be absent in human neurulation [10], suggesting that neural tube closure may follow a somewhat different process in humans [3].

Secondly, many gene-specific homozygous null mouse embryos exhibit additional phenotypes besides NTDs, such as prenatally lethal heart defect. Such syndrome-like examples do not appear particularly close to the models of human NTDs [3]. Also, some mutations may be lethal to human fetus such as Vangl2 [100]. As a result, those embryonic lethal cases are unlikely to become the subject of successful studies.

Thirdly, detailed analysis of a few of the mouse mutants suggests that isolated NTDs can also result from the effect of hypomorphic alleles, combinations of heterozygous mutations, genetic background effects and/or gene-environmental interactions. This partial loss of function or multi-factorial etiologies may more closely resemble to human NTDs [3].

### Outlook

Kibar and colleagues have identified three VANGL1 mutations (V239I, R274Q, and M328T) in patients with sporadic and familial neural-tube defects [101]. However, the phenotype associated with V239I varied among patients. Notably, the mother of the proband with the V239I de novo mutation did not have NTD. They think this finding is consistent with the proposed multi-factorial model for NTD formation. V239I has probably a partial or complete loss of function effect and it interacts with other genetic loci or unknown environmental factors to modulate the incidence and severity of the defect [101].

As discussed above, the development of NTDs is associated with multi-factors. To date, the concept is commonly accepted that the development of NTDs is related to the gene mutations and the gene interaction with other environment factors, which can explain some inexplicable phenomena related to deficiency [136], inositol [25,137], diabetes [138], We think that the gene-environmental interaction is an important process in which the environmental factors can affect the gene expression and affect the process of transcription and translation.

## Conclusions

In this paper, we have reviewed recent studies and highlighted an intimate relationship between PCP signaling pathway and the development of NTDs. The nature of this relationship remains to be further studied. What is certain is that the PCP, also called tissue polarity, is not only restricted to epithelial tissues, but is also found in mesenchymal cells throughout animal development. The PCP signaling pathway is highly conserved in various species, which mediates changes in cell polarity and cell motility in neurulation, through the activation of cytoskeletal pathways, such as RhoA and Rho kinase. Several components of the PCP pathway are expressed in the process of neural tube closure, and the disrupted function of the PCP pathway members in Xenopus, zebrafish and mouse are connected with various defects, and final lead to NTDs. In this process, the interaction of proteins within PCP pathway and PCP proteins with proteins in other pathways are also demonstrated. Although gene mutations in PCP that cause NTDs in humans are rarely reported, it is noted that environmental factors and other genetic factors may affect the expression of the PCP genes.

## Competing interests disclosure

The authors declare that they have no competing interests.

## Authors' contributions

GW was participated in data and information collection and part of the writing.

XH performed part of text writing and the editing of the whole manuscript.

YH wrote the part of the manuscript and information collection. DM was in charge of the whole project and participated in the manuscript writing. All authors read and approved the final manuscript.
